# Loss of action-related function and connectivity in the blind extrastriate body area

**DOI:** 10.3389/fnins.2023.973525

**Published:** 2023-03-09

**Authors:** Or Yizhar, Zohar Tal, Amir Amedi

**Affiliations:** ^1^Department of Cognitive and Brain Sciences, The Hebrew University of Jerusalem, Jerusalem, Israel; ^2^Ivcher School of Psychology, The Institute for Brain, Mind and Technology, Reichman University, Herzliya, Israel; ^3^Research Group Adaptive Memory and Decision Making, Max Planck Institute for Human Development, Berlin, Germany; ^4^Faculty of Psychology and Educational Sciences, University of Coimbra, Coimbra, Portugal; ^5^The Ruth & Meir Rosenthal Brain Imaging Center, Reichman University, Herzliya, Israel

**Keywords:** congenital blindness, neuroimaging, body representation, resting-state fMRI, visuomotor interactions, extrastriate body area, plasticity

## Abstract

The Extrastriate Body Area (EBA) participates in the visual perception and motor actions of body parts. We recently showed that EBA’s perceptual function develops independently of visual experience, responding to stimuli with body-part information in a supramodal fashion. However, it is still unclear if the EBA similarly maintains its action-related function. Here, we used fMRI to study motor-evoked responses and connectivity patterns in the congenitally blind brain. We found that, unlike the case of perception, EBA does not develop an action-related response without visual experience. In addition, we show that congenital blindness alters EBA’s connectivity profile in a counterintuitive way—functional connectivity with sensorimotor cortices dramatically decreases, whereas connectivity with perception-related visual occipital cortices remains high. To the best of our knowledge, we show for the first time that action-related functions and connectivity in the visual cortex could be contingent on visuomotor experience. We further discuss the role of the EBA within the context of visuomotor control and predictive coding theory.

## Introduction

Visual processing in the brain features two pathways, the ventral-occipitotemporal “what” stream and the dorsal-occipitoparietal “where and how” stream (Goodale and Milner, 1992). For many years, researchers assumed that visual streams develop only during specific time windows of critical periods (Hensch, 2004; Knudsen, 2004). However, there is accumulating evidence that the two streams maintain some level of functional selectivity and connectivity independent of visual experience (Striem-Amit et al., 2012b; Fine and Park, 2018; Heimler and Amedi, 2020). Studies of congenitally blind individuals show that category-selective loci operate in a sensory-independent manner (Reich et al., 2012; Heimler et al., 2015) and respond to task-specific information derived from other sensory modalities (Amedi et al., 2002, 2007; Pietrini et al., 2004; Ptito et al., 2005; Poirier et al., 2006; Renier et al., 2010; Sani et al., 2010; Collignon et al., 2011; Reich et al., 2011; Striem-Amit et al., 2012a; Strnad et al., 2013; Striem-Amit and Amedi, 2014; Abboud et al., 2015; Sigalov et al., 2016; Mattioni et al., 2020). Category-specific brain areas can thus form without visual experience in critical periods–the ideal time frames for development in infancy (Kupers and Ptito, 2014; Heimler and Amedi, 2020; Mattioni et al., 2020). For differences in the organization of higher cognitive functions between blind and sighted individuals, see (Amedi et al., 2003, 2004; Bedny, 2017).

These studies explored the stability of perceptual processing in the Occipital lobe. Yet, recent works on sighted individuals show that motor planning and execution can recruit visual cortical areas (Dinstein et al., 2007; Orlov et al., 2010; Lingnau and Downing, 2015; Gallivan et al., 2016). In particular, multiple studies reported that motor actions evoke a neural response in the visual Extrastriate Body Area (EBA) (Astafiev et al., 2004; Peelen and Downing, 2005; Kühn et al., 2011; Gallivan et al., 2013, 2016; Limanowski and Blankenburg, 2016; Zimmermann et al., 2016; Limanowski et al., 2017). Situated in the Lateral Occipital Temporal Cortex (LOTC) and connected to both visual streams, the EBA specializes in the perceptual processing of body parts (Downing, 2001). Even so, evidence from an array of studies finds that both seen and unseen movements recruit the EBA (Astafiev et al., 2004; Peelen and Downing, 2005; Limanowski et al., 2017) and that EBA’s neural activity patterns can decode future motor actions (Kühn et al., 2011; Gallivan et al., 2013, 2016; Orgs et al., 2016). In amputees, the strength of functional connectivity between EBA and sensorimotor areas correlates with higher prosthesis usage (Van Den Heiligenberg et al., 2018). EBA is also active during the rubber-hand illusion and even passive arm movements (Gentile et al., 2015; Limanowski and Blankenburg, 2016; Limanowski et al., 2017), which suggests it is a target for proprioceptive as well as visual information. Although its location is remote from primary Sensorimotor cortices, it appears to play an integral functional part in motor actions, perhaps even independent of vision. Given the now-known task-selective sensory-independent nature of the visual streams, does the EBA develop its action-related functions regardless of visual and motor experience? And do perception and action follow the same developmental constraints?

In a previous study, we used a visual-to-auditory Sensory Substitution Device (SSD) to show that the EBA of blind individuals responds preferentially to stimuli with body-related information (Striem-Amit and Amedi, 2014). The response had a high degree of anatomical consistency across blind individuals, which overlapped with responses elicited by visual body-part images in sighted individuals (Striem-Amit and Amedi, 2014). Furthermore, the blind EBA preserves its functional connections to visual cortices, like the Ventral and Dorsal streams (Striem-Amit and Amedi, 2014). Other studies on the blind brain report maintenance of large-scale functional and structural organization within visual cortices (Striem-Amit et al., 2012b,^2015^; Butt et al., 2013; Burton et al., 2014; Heine et al., 2015; Fine and Park, 2018). Moreover, as the principal role of the EBA is perceptual, one could predict that the lack of visual experience would cause even lower damage to EBA’s action-related functions. Findings from sighted individuals support this prediction. The EBA is also active when visual information is absent, and researchers can decode future body postures from its activity (van Nuenen et al., 2012; Zimmermann et al., 2016, 2018). These results led some researchers to infer that EBA takes part in motor planning by receiving information on the current postural schema and determining a future postural configuration. Thus, we hypothesized that the lack of visual experience does not affect EBA’s motor-related neural responses.

An opposing view is that EBA integrates visuomotor information, and its development is contingent on visual experience. Although much of the blind Visual cortex maintains its intrinsic large-scale organization (Striem-Amit et al., 2012b,^2015^), its functional connections to frontal or sensorimotor cortices decrease (Burton et al., 2014; Heine et al., 2015; Wang et al., 2015; Bauer et al., 2017). In particular, the blind EBA seems to lack significant functional connections to somatosensory and motor cortices (Striem-Amit and Amedi, 2014), in stark contrast to the widespread and pronounced connectivity patterns common for EBA in the sighted (Zimmermann et al., 2018). While EBA retains its perceptual function, the lack of functional connections to sensorimotor cortices hints at a loss of action-related function. Thus, an alternative hypothesis could posit that EBA relies on visual information for actions, and its development is contingent on visual experience. One option is that rather than engaging in motor planning, EBA’s motor-evoked response is part of a perceptual process that anticipates the sensory consequences of motor actions (Astafiev et al., 2004; Orlov et al., 2010; Gallivan et al., 2013). EBA integrates information from upcoming motor actions with incoming visual and proprioceptive sensations to encode limb position (Gallivan et al., 2013, 2016; Gallivan and Culham, 2015; Limanowski and Blankenburg, 2016). Such a dynamic visuomotor representation is redundant for blind individuals and thus would not evolve without visual experience.

Here, we study the effects of congenital blindness on the action-related function of the EBA with task-related and resting-state fMRI (functional Magnetic Resonance Imaging). We expand on our previous study by adapting a cortical parcellation and examining the seed-to-seed functional connections of the EBA to visual and motor regions of interest. Further, we compared the congenitally blind results to a group of sighted participants, where we expect EBA activity during motor tasks. We interpret and discuss our results in the context of the plasticity of action-related functions in visual cortices and their dependency on visual experience.

## Materials and methods

### Participant details

The study included 13 congenitally blind participants. Nine participants participated in the resting-state experiment [eight right-hand dominants, six females, age: 34.5 ± 6.2 (mean ± standard deviation)], and eight participated in the motor experiment (seven right-hand dominants, five females, age: 33.3 ± 9.1). Four blind participants took part in both experiments (see Supplementary Table 1 for further details). We also recruited 29 sighted participants, 20 participated in the resting state experiment (18 right-hand dominants, 12 females, age: 29.5 ± 4.1), and nine participated in the motor experiment (eight right-hand dominants, five females, age: 26.3 ± 2.2). We excluded two additional sighted participants from the resting state analysis due to excessive head movement. Participants had no known neurological conditions, and hand dominance was self-reported. The study received full Helsinki Approval from the Hadassah Medical Center at the Hebrew University of Jerusalem. All participants signed a consent form, and blind participants signed with the companion of an impartial witness.

### Experimental paradigm

#### fMRI motor experiment

The motor experiment included three scanning runs of body movements. Participants moved 12 body parts in succession while lying blindfolded in the MRI (Magnetic Resonance Imaging) scanner. To further decrease the effects of light and visual perception, we instructed participants to keep their eyes closed at all times, and we completely darkened the scanner room. The sequence is in line with the homunculus’ spatial representation in primary Sensorimotor cortices (Penfield and Boldrey, 1937; Zeharia et al., 2012). This continuous paradigm is optimal for topographical mapping (Zeharia et al., 2012) and is also suitable for more conventional analyses (Tal et al., 2016, 2017; Hofstetter and Dumoulin, 2021). Each run consisted of six cycles in which participants moved the 12 body parts: right toes, right foot, right arm, right wrist, right fingers, lips, jaw, left fingers, left wrist, left arm, left foot, left toes (for more details, see Supplementary Table 2). Before the start of a cycle, participants heard a 3 s auditory cue with information about the cycle’s direction (i.e., “start right/left”). Each cycle concludes with a rest period of 6–9 s. Three cycles in each run followed this sequence, while the other three cycles had the opposite direction, starting from the left toes and finishing with the right toes (Supplementary Figure 1). In sum, there were 18 trials for each body part. The sound sequences were produced using Adobe Premiere Pro (Adobe Inc.) and presented using Presentation (Neurobehavioral Systems). We instructed participants to execute movements for 3 s with an interstimulus interval of 1.5 s that included an auditory cue for the next body part. Each trial consisted of three body part movements synchronized with auditory metronome beeps. A body-part movement (e.g., flexion and extension of the arm) lasted 1 s, split evenly between the two actions. Before entering the scanner, we trained participants for 30 min to minimize excess movement.

### Neuroimaging data collection

All brain imaging experiments included anatomical and functional scans from a 3T Siemens Skyra scanner using a 32-channel Head Matrix Coil at The Edmond and Lily Safra Center for Brain Sciences (ELSC) Neuroimaging Unit at the Hebrew University of Jerusalem.

#### fMRI motor experiment

The functional scans that included motor tasks used a multi-band (MB) imaging protocol with a factor of four. We collected the data under the following parameters: TR (Time Repetition) = 1,500 ms, TE (Time Echo) = 32.4 ms, FA (Flip Angle) = 78^°^, imaging matrix = 96*96, FOV (Field of View) = 192 mm*192 mm with an in-plane resolution of 2 mm*2 mm. We acquired 72 axial slices with 2 mm thickness and 0 mm gap for full cortex coverage. We omitted the first ten volumes of each functional run to ensure signal stabilization. High-resolution 3D T1-weighted anatomical images were collected using an MP-RAGE sequence with the following parameters: FOV = 256 mm*256 mm, 160 axial slices, TR = 2,300 ms, TE = 2.98 ms, flip angle = 9^°^, with an in-plane resolution of 1 mm*1 mm. In sum, we acquired between 254 and 272 functional volumes in each run.

#### fMRI resting-state experiment

We collected resting-state data under the following parameters: TR = 3,000 ms, TE = 30 ms, FA = 70^°^, imaging matrix = 64*64, FOV = 240 mm*240 mm with an in-plane resolution of 3.75*3.75 mm. We acquired 50 axial slices with 3 mm thickness and 0 mm gap for full cortex coverage. We acquired scans of 200 volumes for each participant in the blind group without a delayed acquisition and omitted the first 20 volumes to ensure magnetization stability. In the sighted group, we acquired 180 volumes per participant with an automatic system for stabilization. We collected High 3D T1–resolution weighted anatomical images using an MP-RAGE sequence with the following parameters: FOV = 256 mm*256 mm, 160 axial slices, with an in-plane resolution of 1 mm*1 mm.

### Preprocessing

We analyzed data from both experiments using the BrainVoyager 20.6 software package (Brain Innovation, Maastricht). We corrected functional MRI data for slice timing (temporal interpolation to the middle slice of each functional volume), corrected for head motion (trilinear interpolation for detection), and applied a high-pass filter (cut-off frequency of two cycles per run). None of the scans included during the two experiments showed translational motion exceeding a maximum of 2 mm displacement or a maximum rotational motion exceeding 2^°^ in any direction. We normalized anatomical T1 scans of each subject to a standardized MNI (Montreal Neurological Institute) space (ICBM-152). We then applied the calculated normalization parameters to all the subject’s functional scans. Cortical reconstruction included the segmentation of white matter using a grow-region function. The remaining cortical surface was then inflated and aligned to a 3D cortical reconstruction of an MNI-normalized brain (FreeSurfer’s fsAverage brain).

#### fMRI motor experiment

We spatially smoothed the data from the motor task runs (spatial Gaussian smoothing, Full Width at Half Maximum = 6 mm) to overcome inter-subject anatomical variability.

#### fMRI resting-state experiment

Functional resting-state data did not undergo spatial smoothing to avoid false correlations.

### Cortex-based alignment

To acquire precise anatomical markers as Regions of Interest (ROI), we used a cortex-based alignment (CBA) algorithm implemented in BrainVoyager (Frost and Goebel, 2012) and aligned the hemispheres of all participants to a fsAverage brain surface. In short, the process morphs reconstructed and folded hemispheres into a sphere that produces a curvature map with differing degrees of smoothness. The alignment itself is an iterative procedure that follows a coarse-to-fine matching strategy to a target brain that gradually decreases the smoothness of the sphere’s surface. Importantly, we merged the aligned hemispheres to create a bilateral cortical alignment, rather than the more common procedure of aligning and analyzing each hemisphere in isolation. Using the alignment, we transformed all the functional datasets from a standard MR volume space to the target surface. This procedure enabled us to execute whole-brain analyses that produce statistical maps on both cortical hemispheres.

### Parcellation and ROI selection

As some of our participants are blind, precise functional identification of the EBA was impossible on a subject level-basis. To identify the EBA and other relevant ROIs, we used anatomical landmarks delineated from the cortical parcellation suggested by Glasser et al. (2016). The parcellation considers structural and functional data collected in the Human Connectome Project (HCP). In localizing EBA, we used data provided by the HCP (van Essen et al., 2017) to identify cortical areas that preferentially respond to images of body parts compared to images of tools, faces, and lpaces (Glasser et al., 2016). We focused our analysis on the MT complex, a known location of the EBA (Ferri et al., 2013).

#### fMRI resting-state experiment

For the functional connectivity analysis, we selected eight ROIs (three for visual areas and five for sensory-motor areas) in addition to EBA. We chose visual regions associated with EBA’s functions, the visual Ventral and Dorsal streams, and the primary Visual cortex (V1). For sensorimotor ROIs, we chose areas that produce consistent activity during motor planning and action, or process incoming proprioceptive inputs. These include the Premotor cortex (PMc), the primary Motor cortex (M1), the primary Somatosensory cortex (S1), the Operculum and Insular cortex (Operculum), and the Supplementary motor Area along with adjacent cingulate motor areas (SMA). For each ROI, we chose the corresponding parcellations from HCP-MMP1.0 (Glasser et al., 2016). A description of the ROIs and the cytoarchitecture regions that comprise them are in Supplementary Table 3. Following the fMRI motor experiment, we wanted to use a bilateral EBA seed and bilateral visual and motor ROIs. To establish that left and right EBAs have similar connectivity patterns, we analyzed the functional connections between left-lateralized EBA and right-lateralized ROIs, as well as between right-lateralized EBA and left-lateralized ROIs in the sighted group (Supplementary Figure 2).

### GLM analyses

We performed our analyses on the reconstructed and inflated fsAverage brain surface to which we aligned all participants’ cortices with cortex-based alignment.

#### fMRI motor experiment

We first computed statistical parametric maps from a single-subject General Linear Model (GLM). We chunked the body part movements into five predictors of major anatomical segments: right foot (toes and foot), right hand (digits, arm, and wrist), face (jaw and lips), left hand (digits, arm, and wrist), left foot (toes and foot). In our model, all body parts within each group are explained by the same predictor (e.g., “face” for jaw and lips). The model predictors were convoluted with a canonical Hemodynamic Response Function. Before fitting the GLM model, time courses of the preprocessed data underwent z-normalization. For the group-level analyses, we used a multi-level random-effects model. The statistical threshold criterion for individual vertices was *p* < 0.05. We corrected all the results for multiple comparisons using a cluster-size threshold estimator based on a Monte Carlo simulation approach with 500 iterations, as implemented in BrainVoyager 20.6. We conducted ROI analyses of the EBA by contrasting each of the five body part groups against a baseline parameter of zero (i.e., no response). We executed all these analyses using MATLAB software (MathWorks). ROI statistical tests were double-sided and corrected for multiple comparisons using False Discovery Rate (FDR, α = 0.05). We also examined if between-group differences in EBA’s motor-evoked response are bilateral and are anatomically consistent for left-and right-hand movements. We plotted the peak activity for the sighted group’s right-hand movements within bilateral EBA. We defined a seed that includes all vertices within a 4 mm distance from the peak group activity (Supplementary Figure 3). To establish bilaterality, we analyzed the activity within that seed for left-hand movements (see Supplementary Figure 4) for a magnified view of motor-evoked response around EBA).

#### fMRI resting-state experiment

To describe the EBA connectivity patterns, we extracted the EBA signal time course by averaging the data across all EBA vertices at each time point. Next, we extracted the MR signal from the ventricles and subarachnoid spaces (i.e., Cerebrospinal Fluid). We wanted to use the signal as a confound in our model, as these voxels include noise factors such as global signal drift. We used an embedded function found in BrainVoygaer to remove skull tissue, head tissue and subcortical structures. From the resulting image, we created a mask of voxels with intensity levels below the threshold for white and gray matter segmentation. We repeated this procedure in each participant’s brain following MNI normalization. Similar to the EBA, we averaged the time course across all voxels at each time point. Time-course samples were then z-normalized and modeled as predictors in a first-level GLM connectivity analysis - the EBA time course as an explanatory predictor and the Cerebrospinal Fluid (CSF) time courses as confounding predictors. In the group random effect analyses, we used beta images from first-level GLM analyses and treated the participants as a random factor. First, we performed a whole-brain analysis by contrasting connectivity estimates from both EBAs against the baseline. The vertex-wise statistical threshold for the whole-brain analysis was *p* < 0.05, corrected for multiple comparisons using a Monte Carlo permutation test. Next, we performed seed-to-seed ROI analyses from the EBA to five sensorimotor and three visual cortical areas (see ROI selection). As motor movements recruit both EBAs, we inspected the connectivity between bilateral ROIs and EBA predictors. The statistical threshold was *p* < 0.05, corrected for multiple comparisons with a False Detection Rate (α = 0.05).

## Results

To explore the effects of visual experience on EBA’s action-related function, we trained sighted and congenitally blind participants to move 12 unilateral body parts in search of motor-evoked responses. In a separate experiment, we investigated EBA connectivity patterns to sensorimotor and visual loci during rest. To characterize the whole-brain activity and functional connectivity, we used a Cortex Based Alignment technique (Frost and Goebel, 2012) that merges both hemispheres (see methods). We performed all group-level random-effects analyses on this aligned brain.

### The blind EBA does not respond to active movements

**FIGURE 1:**
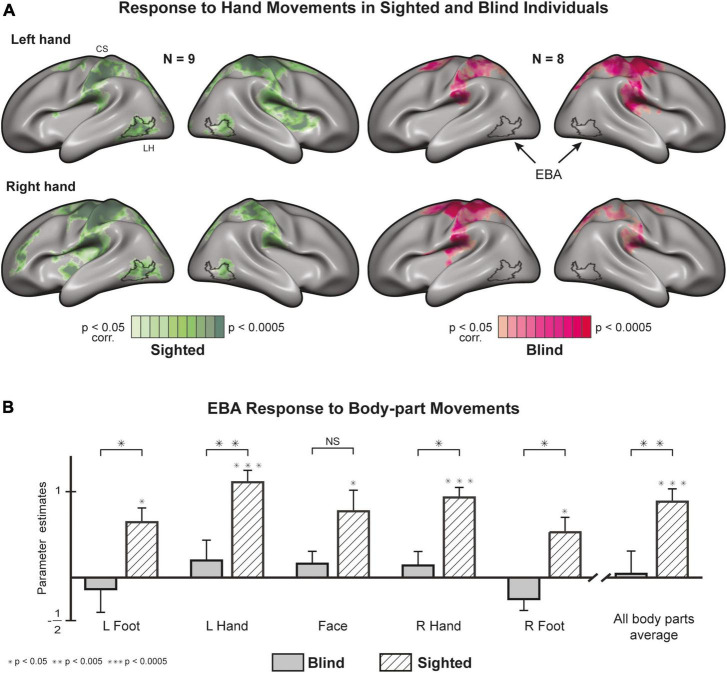
Motor-evoked responses to body part movements (whole-brain and EBA localizer). **(A)** Whole-brain statistical parametric maps of hand movements, most commonly associated with EBA activity, plotted on both cortical hemispheres after cortex-based alignment [random effects General Linear Model (GLM)]. Hand movements recruit EBA in sighted individuals but not in blind individuals (see Supplementary Figure 5 for other body parts). EBA, Extrastriate Body Area; CS, central sulcus. **(B)** Regions of Interest (ROI) analysis of EBA response to various body parts. The region is significantly active in the sighted and not in the blind. A between-group analysis (random effects GLM, *n* = 17) reveals significant differences in the response to movements of the right foot, right hand, left foot, and left hand (for results in separate hemispheres, see Supplementary Figure 6).

A whole-brain GLM analysis in sighted individuals shows robust activations in and around bilateral EBA (Figure 1A) for hand movements (see Supplementary Figure 5 for similar results of feet and face). In the blind group, we did not find activity in or around EBA for all unilateral movements. Hand movements in both groups result in localized activity in somatosensory and motor areas, suggesting a limited whole-brain noise effect. An ROI analysis of motor-evoked responses (Figure 1B) in EBA confirmed the previous observations, with statistically significant responses in the sighted and insignificant responses in the blind (Supplementary Table 4). An analysis of group differences showed that the sighted group had significantly stronger EBA responses for the left hand [t (15) = 3.39, *p* = 0.004, *d* = 1.52] and right hand [t (15) = 2.44, *p* = 0.027, *d* = 1.08], as well as the left foot [t (15) = 2.81, *p* = 0.013, *d* = 1.26] and right foot [t (15) = 2.54, *p* = 0.022, *d* = 1.14]. We did not find a significant difference between the groups in the face condition [t (15) = 1.66, *p* = 0.118].

### M1 responses are comparable between blind and sighted individuals

**FIGURE 2:**
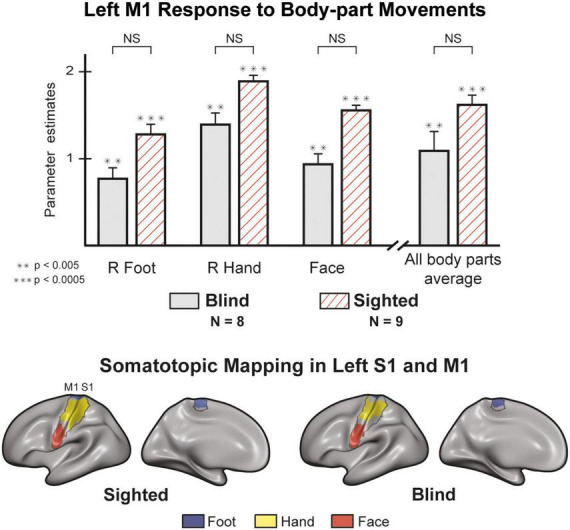
M1 responses are comparable between blind and sighted individuals [random effects General Linear Model (GLM)]. There were no significant group differences in M1 activity (left hemisphere) for all unilateral and face movements (Supplementary Table 5). Regions of Interest (ROI) analysis of right M1 produced similar results (Supplementary Figure 7). The sighted and blind groups show similar somatotopic organization in S1 and M1. We created the maps by contrasting each condition with all others. We then plotted and colored all statistically significant vertices (*p* < 0.05, corrected). The results on the left hemisphere represent the response to right hand, right foot, and face movements. We found similar results in the right hemisphere for contralateral effectors (Supplementary Figure 5). Error bars are for the standard error. Large asterisks indicate between-group significance, small asterisks indicate within-group significance against the baseline. NS, non-significant.

An ROI analysis (Figure 2) of the Left primary Motor cortex (M1) shows no differences between the blind and sighted for movements of the right hand [t (15) = 1.519, *p* = 0.15] or right foot [t (15) = 1.709, *p* = 0.108]. The statistical difference between the groups for face movement did not survive a multiple comparison correction [t (15) = 1.28, *p* = 0.04]. Analysis of motor-evoked responses in Right M1 shows congruent results, with no significant differences between the groups (Supplementary Table 5). A mixed design ANOVA with a within-factor of body-part and a between-factor of the group found no differences in the group responses [F (1,135) = 3.619, *p* = 0.077, η^2^ = 0.07] and no interaction between group and body-part [F (7,135) = 0.802, *p* = 0.587, η^2^ = 0.016]. We also looked at the selectivity of neural responses in S1 and M1 (Figure 2). In both groups, we observed activity that follows a topographic organization (see Supplementary Figure 7 for the right hemisphere), in line with the canonical Penfield homunculus (Penfield and Boldrey, 1937; Zeharia et al., 2012).

### Congenital blindness drastically alters EBA’s connectivity

**FIGURE 3:**
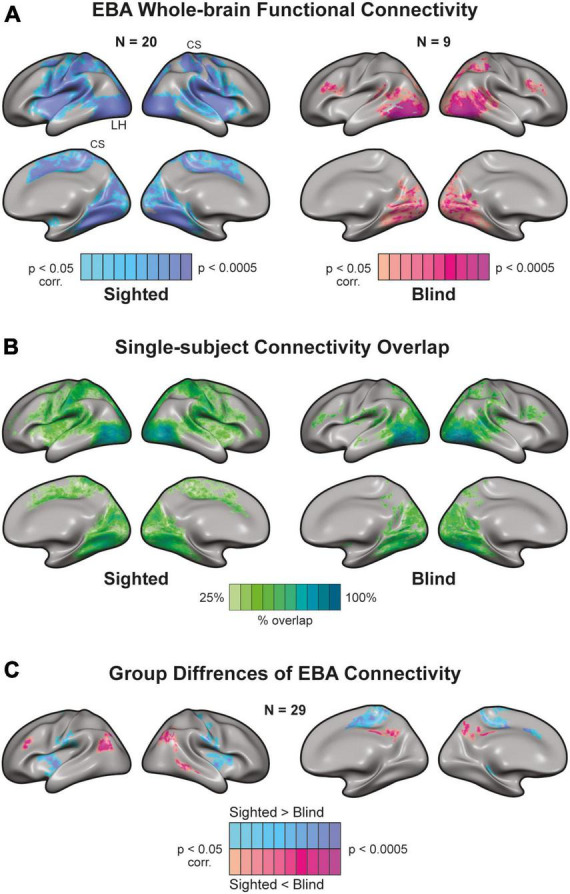
Whole-brain connectivity of Extrastriate Body Area (EBA) seed in blind and sighted individuals [random effects General Linear Model (GLM)]. **(A)** There is a high functional connection between the EBA [independent localizer from Human Connectome Project (HCP) atlas] and visual regions in sighted and blind individuals. However, EBA connectivity to sensorimotor areas exhibits a strikingly different picture. EBA has widespread connectivity to sensorimotor cortices in the sighted. Yet, there is a disconnection between EBA and sensorimotor regions in the congenitally blind. CS, central sulcus. **(B)** Single-subject connectivity overlap in sighted and blind groups. We created the probabilistic maps from single-subject EBA connectivity results. Connectivity to visual areas was consistent across blind and sighted participants. In addition, there was a connectivity overlap in the sighted around S1, M1, and associative sensorimotor cortices. **(C)** Group differences of EBA connectivity. The sighted had higher connectivity around sensorimotor regions and weaker connectivity around the Precuneus and lateral Parietal cortex.

We used resting-state fMRI data to examine the effects of visual development on EBA’s connectivity to somatosensory and motor cortices. Under the assumption that neurons that fire together wire together, neural activity at rest should be reflective of activity during tasks. As such, neurons that are functionally co-activated should also have correlated activity during rest. To examine these connections, we extracted the resting-state time course of the EBA from each subject and used these as predictors in our second-level analysis. Given the bilateral nature of motor-related activity in the EBA, we defined the left and right EBA as a combined seed and analyzed their connectivity to the whole brain. To verify this assumption, we mapped the functional connectivity between the sighted EBA (left and right) and contralateral visual and motor ROIs. We found support for our assumption in comparable patterns of contralateral connectivity for left-lateralized and right-lateralized EBAs (Supplementary Figure 2). Both groups exhibited known connectivity patterns (Striem-Amit and Amedi, 2014; Zimmermann et al., 2018) to visual brain areas around the Occipital lobe (Figure 3A). EBA connectivity in the sighted group was more widespread. We found prominent connections to M1, S1, Inferior Parietal Lobule, and associative areas such as the Operculum. Next, we used probabilistic mapping to observe the consistency of connectivity patterns within the blind and sighted groups. We computed single-subject connectivity maps (corrected for multiple comparisons) and calculated the ratio of cross-subject overlap for each vertex on the cortex (Figure 3B). Both groups had a high degree of connectivity overlap in cortical visual areas, while sighted individuals also exhibited some cross-subject overlap around primary Somatosensory and Motor cortices. There was significantly higher connectivity (Figure 3C) in the sighted group around the Pre-and-post Central gyri, Operculum, Insula, Cingulate, and Superior Parietal Lobule (Glasser et al., 2016). EBA of the blind group had stronger connections with the Inferior-lateral Parietal cortex, Lateral Prefrontal cortex, and Precuneus. These areas are canonical to the brain’s Default Mode Network (DMN), active during non-task states (Raichle, 2015).

### There is a functional disconnection between the blind EBA and sensorimotor cortices

**FIGURE 4:**
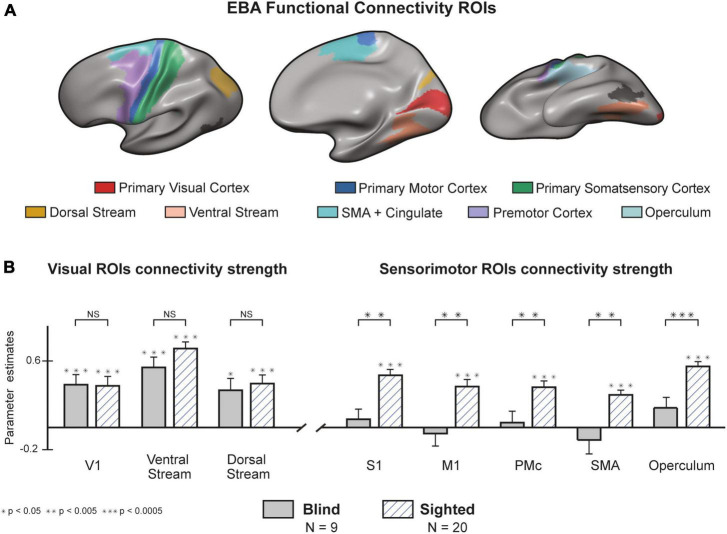
There is a functional disconnection between the blind Extrastriate Body Area (EBA) and sensorimotor cortices. **(A)** Regions of Interest (ROIs) from the Human Connectome Project (HCP) atlas (Glasser et al., 2016). A full cytoarchitectural breakdown of each area is in Supplementary Table 3. **(B)** Congenital blindness alters EBA’s connectivity profile. Seed-to-seed analysis of connectivity strength from the EBA to sensorimotor and visual seeds. In both groups, resting-state connectivity to visual cortices was significantly higher than the baseline. Connectivity to sensorimotor regions was significantly higher in the sighted compared to the blind group (random effects GLM, *n* = 29). We found similar results when analyzing the connectivity from EBA to the right and left hemispheres separately (Supplementary Figure 8). Error bars are for the standard error. Large asterisks indicate between-group significance, small asterisks indicate within-group significance against the baseline. NS, non-significant.

In addition to our whole-brain analysis, we wanted to classify the connectivity strength of the EBA with three visual and five sensorimotor cortical regions (Supplementary Table 6). We based the categorization of ROIs on the parcellation offered by Glasser et al. (2016) (Figure 4A, See methods and Supplementary Table 3 for detailed description). both groups showed a significant interaction between the EBA and cortical visual areas (Figure 4B). no statistical differences were found between the groups in the connectivity strength to the primary visual cortex [t (27) = 0.073, *p* = 0.942], or to the visual ventral stream [t (27) = 1.514, *p* = 0.141] and dorsal stream [t (27) = 0.41, *p* = 0.685]. In stunning contrast, the strength of connectivity to sensorimotor areas was significant in the sighted group and insignificant in the blind group. These findings show that blind participants preserve the intrinsic connectivity of the EBA to visual cortical perceptual areas, while on the other hand, there is a significant decrease in the extrinsic connectivity to sensorimotor cortices. In a direct group comparison, the sighted group showed stronger connectivity between the EBA and the primary motor cortex [t (27) = 3.376, *p* = 0.002, *d* = 1.07], primary somatosensory cortex [t (27) = 3.79, *p* < 0.001, *d* = 1.28], Supplementary motor area [t (27) = 3.796, *p* < 0.001, *d* = 1.27], premotor cortex [t (27) = 2.91, *p* = 0.007, *d* = 0.58], and operculum [t (27) = 3.881, *p* < 0.001, *d* = 1.42].

## Discussion

We studied the action-related function and connectivity of the EBA in both congenitally blind and healthy-sighted adults. We replicated previous results in the sighted and found that the EBA is indeed active during unseen motor actions of the hands and feet. However, the picture was completely different for the blind group, where the EBA did not show a significant response to any body part movements, a sharp contrast to its sensory-independent behavior in perceptual tasks (Heimler and Amedi, 2020). Our analysis of resting-state functional data revealed significant connectivity between EBA and visual cortices in both groups, but a decrease in connectivity between the blind EBA and sensorimotor regions compared to the sighted.

In a previous study, we showed that blind individuals’ perception of body-related stimuli results in consistent activity in the EBA (Striem-Amit and Amedi, 2014). Other studies report a similar pattern for other category-selective areas in the visual Ventral and Dorsal streams (Pietrini et al., 2004; Ptito et al., 2005; Poirier et al., 2006; Amedi et al., 2007; Sani et al., 2010; Reich et al., 2011; Abboud et al., 2015). These areas display supramodal behavior, where a specific task defines their function (e.g., perceiving a stimulus with body-related information) and not the input modality (Pascual-Leone and Hamilton, 2001; Heimler et al., 2015; Cecchetti et al., 2016; Hurka et al., 2017; Fine and Park, 2018; Heimler and Amedi, 2020). Here, we found that this supramodal principle does not apply to action-related activity in the EBA. Body-part movements did not activate the EBA in the blind group, even for hand movements that elicit robust activity in the sighted. Additionally, the functional connectivity analysis showed that blind individuals have weaker connections between the EBA and sensorimotor areas, but the blind EBA maintain its internal connectivity profile to other visual cortices. Other supporting findings indicate that the deprived visual cortex of blind individuals maintains its large-scale intrinsic organization (Striem-Amit et al., 2012b,^2015^; Butt et al., 2013; Burton et al., 2014; Heine et al., 2015; Bauer et al., 2017), coupled with a decrease in connectivity between visual and sensorimotor cortices (Burton et al., 2014; Striem-Amit and Amedi, 2014; Heine et al., 2015; Bauer et al., 2017).

The discrepancy in the EBA connectivity profile is, at first impression, a little counterintuitive. One might expect that visual deprivation results in weaker functional connections with visual cortices and stable functional connections with non-visual cortices. This assumption would also be consistent with previous findings of neural responses in the blind visual cortex to high-order tasks (Amedi et al., 2003, 2004; Bedny et al., 2011; Bedny, 2017). Instead, we found an absence of connectivity between the EBA and non-visual cortices. In turn, the maintenance of perception-related functions explains the stability within the visual system. A possible interpretation is that EBA engages in visuomotor computations (Orlov et al., 2010) that we need during typical sensory and motor development but are of no use for blind individuals. Differently, Ventral stream areas could have a computational bias for perception-related tasks and even respond to non-visual stimuli in healthy-sighted individuals (Amedi et al., 2001; Sathian, 2005; Lacey et al., 2009; Debowska et al., 2016; Siuda-Krzywicka et al., 2016; Tal et al., 2016; Bola et al., 2017). The differences observed here also point to the source of incoming signals to the EBA during action-related processing. While it is well established that EBA receives visual inputs from the primary Visual cortex via feedforward connections (Lamme and Roelfsema, 2000), the source of neural inputs during motor actions is less clear. One suggestion is that motor-related information travels through direct connections from the Frontoparietal to the Occipital cortices (Gallivan et al., 2013). Our results point to another option that inputs to the EBA are from Parietal and Premotor structures involved in somatosensory and motor processing (Gallivan et al., 2013, 2016; Lingnau and Downing, 2015; Limanowski and Blankenburg, 2016; Orgs et al., 2016; Simos et al., 2017). These types of lateral, rather than feedforward, connections are less hardwired (Fine and Park, 2018). In the blind brain, the lack of co-occurring inputs from visual and proprioceptive sensations into EBA would erode these weaker connections. Oppositely, EBA maintains its perceptual-related function through feedforward hardwired connections that are the pipeline for perceptual processing (Fine and Park, 2018). These contrasting behaviors also hint at the sensibility of the visual cortex during critical periods. The Occipital lobe might have low sensitivity to visual perceptual experience, which is hardwired, and high sensitivity to visuomotor action-related experience. At the very least, our results show that EBA’s motor recruitment is contingent on visual experience and that its function does not depend only on sensorimotor experience.

How does the lack of EBA response in the blind inform us about EBA’s role in motor-related processing in the sighted? Our findings suggest that EBA holds a whole-body motor representation. In sighted individuals, unilateral movements result in bilateral activations of EBA, especially for hand and foot movements. Unlike the typical contralateral or ipsilateral motor responses, each unilateral EBA computes information about both sides of the body, with an emphasis on the limbs. Moreover, action-related activity in bilateral EBA has been shown to correlate with the usage rate of hand prostheses in unilateral amputates (Van Den Heiligenberg et al., 2018), suggesting a possible connection between EBA function and the perception of limb position. Notably, bilateral EBA is active when participants perceive visual images of body parts (Orlov et al., 2010; Kühn et al., 2011; Gallivan et al., 2013; Zimmermann et al., 2018). These characteristics are beneficial for an EBA that takes part in motor control (Kühn et al., 2011; van Nuenen et al., 2012; Gallivan and Culham, 2015; Zimmermann et al., 2016) and predictive processing of sensory information (Astafiev et al., 2004; Orlov et al., 2010; Gallivan et al., 2013). Under the predictive processing theory, the brain generates representations to predict future sensory inputs (Keller and Mrsic-Flogel, 2018) and continuously compares these predictions to actual sensory inputs. The resulting error updates an internal representation that culminates in perception. EBA’s activity patterns fit this framework, being active during active (Astafiev et al., 2004; Gallivan et al., 2013, 2016; Zimmermann et al., 2016; Limanowski et al., 2017) and passive (Limanowski and Blankenburg, 2016) movements and allowing for the decoding of future postures from its activity during motor planning (Kühn et al., 2011; Gallivan et al., 2013, 2016; Orgs et al., 2016; Zimmermann et al., 2016). Furthermore, visuo-proprioceptive incongruences decrease EBA’s effective connectivity to Parietal cortices (Limanowski and Blankenburg, 2017), which may reflect a breakdown in the prediction process. The internal representation is thus available before and while incoming sensory information reaches the brain (Keller and Mrsic-Flogel, 2018), covering the duration of stimulus expectation and perception. Finally, EBA has strong connections with primary Sensorimotor and associative cortices, which are the source and target of information for the internal model (Keller and Mrsic-Flogel, 2018). Concerning our results, the EBA could provide a multimodal representation that predicts incoming visual information. EBA activity during unseen movement in the sighted reflects the recruitment of large networks that expects incoming sensory information, even if it is not there. In congenital blindness, this internal model becomes redundant as visual information is unavailable.

In conclusion, our findings demonstrate that EBA’s motor-related responses are contingent on visual experience and are absent in congenitally blind individuals. We provide initial steps toward studying plastic changes to action-related functions in the deprived visual cortex. Additionally, this work furthers our understanding of the EBA’s dependence on visual information and experience in sighted individuals.

## Data availability statement

The raw data supporting the conclusions of this article will be made available by the authors, without undue reservation.

## Ethics statement

The studies involving human participants were reviewed and approved by Hadassah Medical Center, the Hebrew University of Jerusalem. The patients/participants provided their written informed consent to participate in this study.

## Author contributions

OY and AA: conceptualization and validation. OY: data curation, formal analysis, methodology, visualization, and writing—original draft. AA: funding acquisition and supervision. OY and ZT: investigation and project administration. OY, ZT, and AA: reviewing, editing, and approving the submitted version.
