# Supplementary material for: Loss of action-related function and connectivity in the blind extrastriate body area
Source: Front Neurosci. 2023 Mar 9;17:973525. doi: 10.3389/fnins.2023.973525 (PMC10035577; doi:10.3389/fnins.2023.973525)

SUPPLEMENTARY INFORMATION

Supplementary Table S1. Characteristics of blind participants in the motor and resting-state experiments.

#	Age	Sex	Handedness	Causes of blindness	Light perception	Experiment	
						Motor	Resting state
1	37	F	Right	Enophthalmos	None	X	X
2	30	M		Persistent Hyperplastic Primary Vitreous		X	
3	28	M		Microphthalmia and retinal detachment		X	
4	30	F	Left			X	X
5	30	F	Right	Retinopathy of prematurity		X	
6	48	M				X	X
7	44	F				X	X
8	20	F				X	
10	29	F					X
11	29	F					X
13	41	M					X
12	33	F		Microphthalmia			X
13	33	F		Leber Congenital Amaurosis	Faint		X

Supplementary Table S2. Descriptions of effector movements in the fMRI motor experiment.

Effector	Body movement	Further details
Toes	Flexion and Extension	Participants moved their toes towards and away from the inner foot
Foot	Inversion and Eversion	Participants moved their foot sideways while abstaining from moving their toes
Arm	Flexion and Extension	Participants moved their arms, bending at the elbow joint (humeroulnar)
Wrist	Flexion and Extension	Participants moved their wrist on the sagittal plane (radiocarpal joint)
Fingers	Flexion and Extension	Participants folded their extended fingers into a fist
Lips	Protraction and Retraction	Participants performed a slight protracted movement, similar to that of a faint kiss
Jaw	Depression and Elevation	Participants slightly moved their jaw up and down, tapping gently their lower and upper teeth

Supplementary Table S3. List of areas selected from the HCP-MMP1.0 parcellation and the cytoarchitectural loci that comprise them. The parcellation of anatomical loci into macroscale areas (e.g., 'Dorsal Visual Stream") can be found in the supplementary material of the publication with the same descriptions (Glasser et al. 2016).

**Area description**		**List of anatomical loci**	**Surface Area (mm^2)**	
			**Left hemisphere**	**Right hemisphere**
**Visual**	Primary Visual Cortex (V1)	V1	739	754
	Dorsal Visual Stream	V3A, V6, V6A, V7, IPS1, V3B	556	615
	Ventral Visual Stream	FFC, VVC, V8, PIT, VMV1, VMV2, VMV3	767	869
**Motor**	Primary Somatosensory Cortex (S1)	1, 2, 3a, 3b	2499	2316
	Primary Motor Cortex (M1)	4	1028	1035
	SMA/Cingulate (SMA)	24dd, 24dv, 6mp, 6ma, SCEF	1360	1337
	Premotor Cortex (PMc)	6a, 6d, FEF, 55b, PEF, 6v, 6r	1354	1398
	Operculum	FOP1, FOP2, FOP3, FOP4, FOP5, 43, OP1, OP2-3, OP4, PFcm	1762	1562
Extrastriate Body Area (EBA)		FST, PH, V4T	314	290

Supplementary Table S4. Random effects GLM results of body-part movements in the EBA for sighted (n = 9) and blind (n = 8) individuals.

	Sighted			***Blind***			***Difference***
	β	*SE*	*p*	β	*SE*	*p*	*p*
Right foot	0.53	0.18	0.02	-0.25	0.28	0.401	0.023
Right Hand	0.93	0.12	<0.001	0.14	0.34	0.69	0.028
Face	0.77	0.25	0.016	0.16	0.30	0.607	0.118
Left Hand	1.11	0.15	<0.001	0.23	0.24	0.386	0.004
Left foot	0.64	0.18	0.006	-0.20	0.28	0.492	0.013

Supplementary Table S5. Random effects GLM results for contralateral body-part movements in left and right Primary Motor Cortices (M1) for sighted (n = 9) and blind (n = 8).

		Sighted			*Blind*			*Difference*
		β	*SE*	*p*	*β*	*SE*	*p*	*p*
Left M1	Right Foot	1.28	0.24	<0.001	0.78	0.26	0.019	0.15
	Right Hand	1.89	0.17	<0.001	1.39	0.27	0.001	0.108
	Face	1.56	0.13	<0.001	0.94	0.27	0.011	0.04*
Right M1	Left Foot	1.23	0.22	<0.001	0.69	0.19	0.008	0.074
	Left Hand	2.19	0.21	<0.001	1.57	0.15	<0.001	0.023*
	Face	1.1	0.19	<0.001	0.74	0.25	0.023	0.239

Supplementary Table S6. Seed-to-seed analysis of connectivity strength from the EBA to bilateral sensorimotor and visual seeds. We calculated a random effects GLM in the blind (n = 9) and sighted (n = 20) using the EBA time-course as a normalized model predictor.

		Sighted			*Blind*			*Difference*
		β	*SE*	*p*	*β*	*SE*	*p*	*p*
**Visual ROIs**	S1	0.47	0.06	<0.001	0.07	0.10	0.483	<0.001
	SMA	0.29	0.05	<0.001	-0.11	0.13	0.416	<0.001
	M1	0.37	0.07	<0.001	-0.05	0.12	0.663	0.002
	Premotor	0.36	0.06	<0.001	0.04	0.11	0.692	0.007
	Operculum	0.52	0.04	<0.001	0.11	0.07	0.1	<0.001
**Sensorimotor ROIs**	Ventral Stream	0.71	0.07	<0.001	0.54	0.10	<0.001	0.142
	Dorsal Stream	0.40	0.09	<0.001	0.34	0.12	0.024	0.685
	V1	0.38	0.09	<0.001	0.39	0.10	<0.001	0.942

Supplementary Figure S1. Experimental design for the motor fMRI scan. Each cycle of body part movements begins with the toes of one side of the body and ends with the toes of the other side. Each run had six such cycles, three starting from the left toes and three from the right toes. Each cycle begins with a three-second auditory cue on the cycle's direction (e.g., "start from the right side"). In every trial, participants moved a body part for three seconds following a 1.5-second auditory cue with the body part name. Over three functional runs, participants performed 18 cycles starting from left or right. A rest period of 6-9 seconds followed each cycle (bottom panel, marked in black).

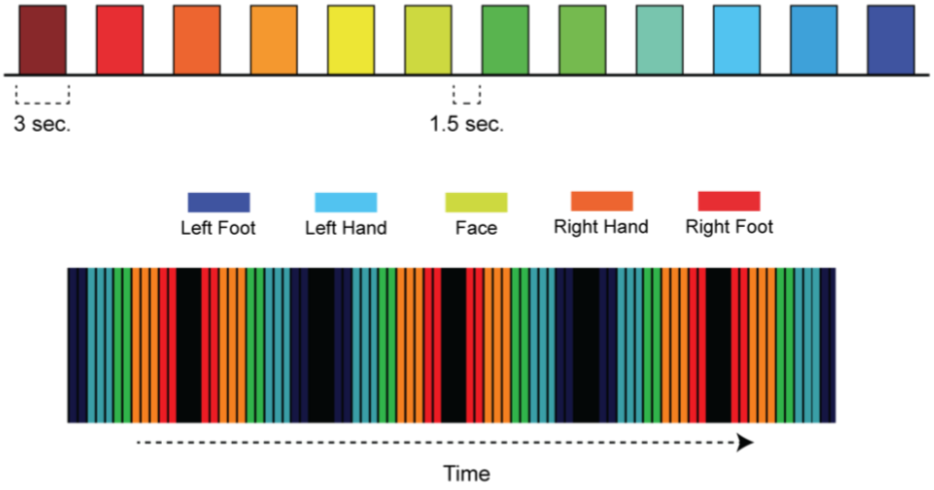

Supplementary Figure S2. Seed-to-seed analysis of cross hemispheric EBA connectivity in the sighted group. Left and right-lateralized EBAs show significant functional connectivity to contralateral visual and motor ROIs. Asterisks indicate within-group significance against the baseline. (* < 0.05, ** < 0.005, *** < 0.0005).

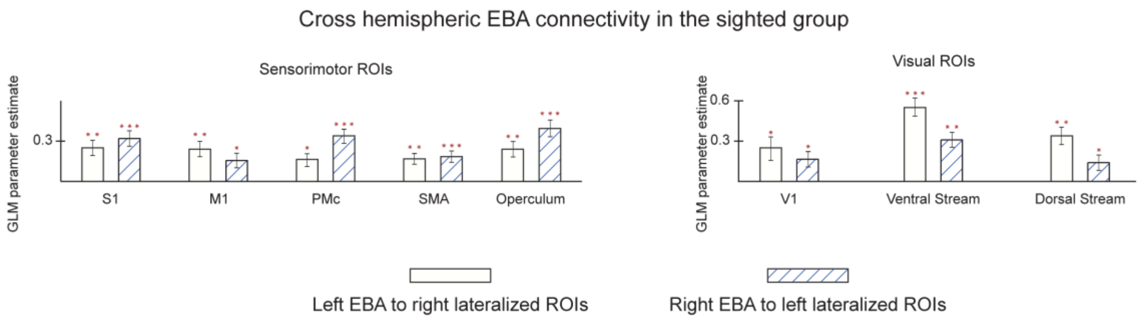

Supplementary Figure S3. ROI analysis of peak activity in the EBA from right-hand movements in the sighted group. We analyzed motor-evoked responses to left-hand movements in the sighted within this seed (n = 9, random effects GLM, $p < 0.05$). Left-hand movements resulted in significant activity ($t(8) = 11.43$, $p < 0.001$, $d = 2.16$), exemplifying the bilateral EBA response to unilateral movements. Error bars represent the standard error.

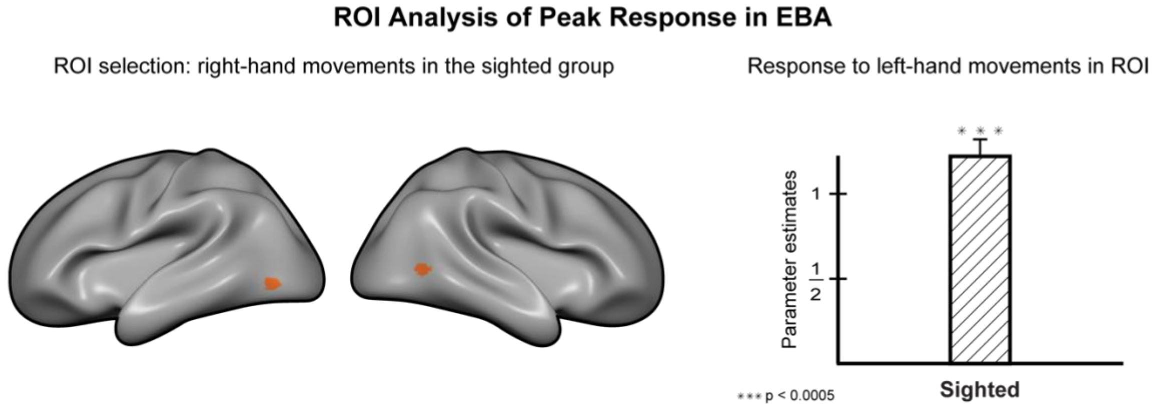

Supplementary Figure S4. Whole-brain statistical parametric maps of hand movements, most commonly associated with EBA activity. The results are plotted on both cortical hemispheres after cortex-based alignment (random effects GLM) with an additional magnified view of the EBA.

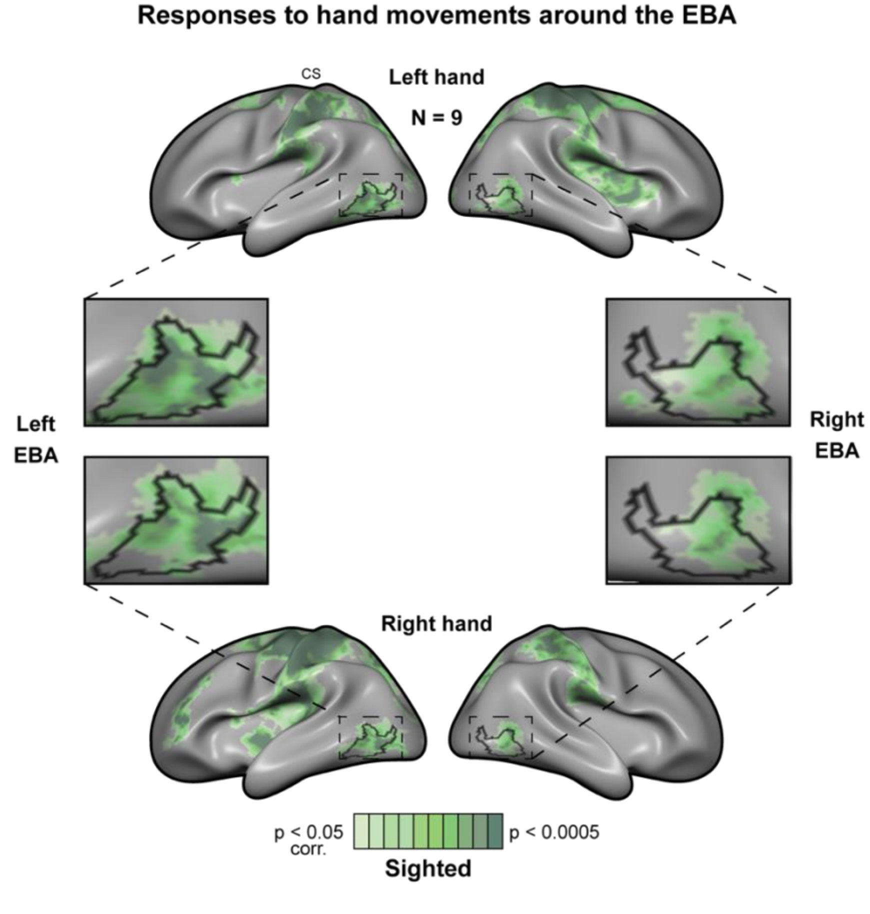

Supplementary Figure S5. Motor-evoked responses to body part movements. Feet and face movements recruit EBA in the sighted but not in the blind. Whole-brain statistical parametric maps are plotted on both cortical hemispheres after cortex-based alignment (random effects GLM). EBA, Extrastriate Body Area. CS, central sulcus.

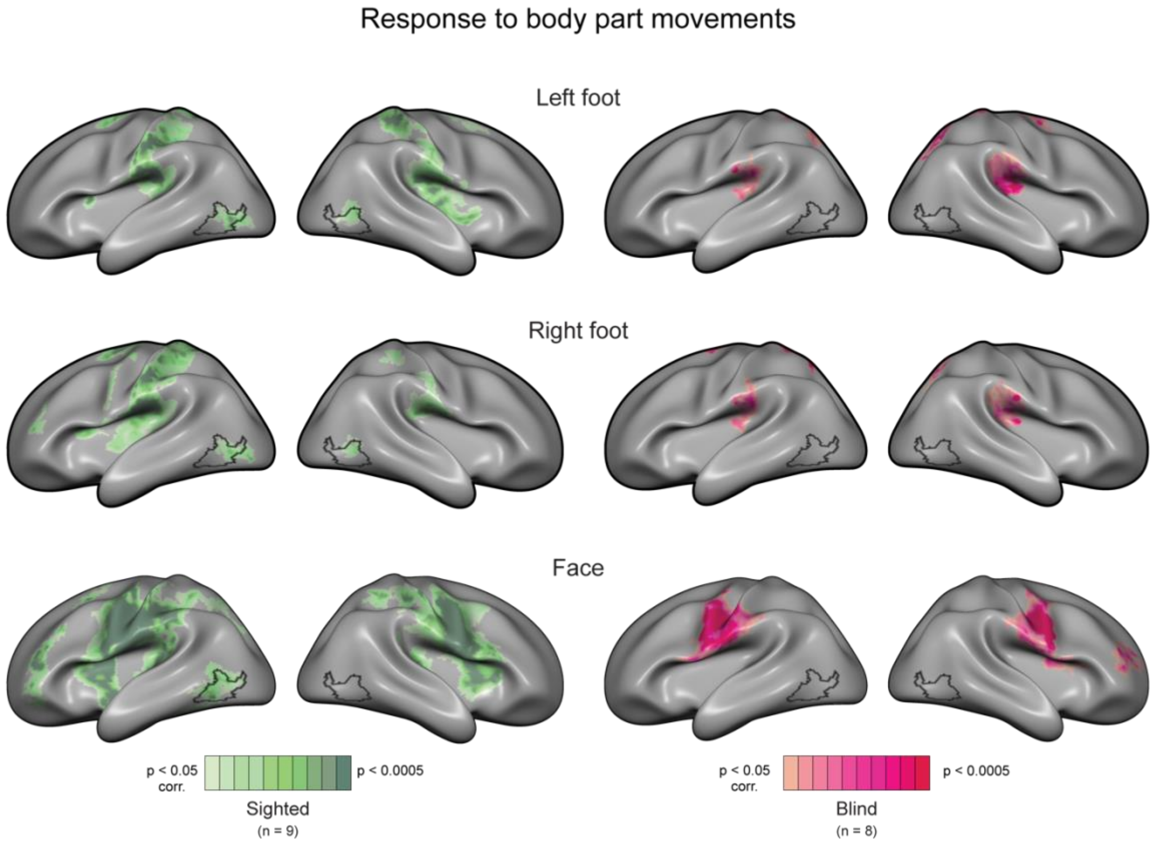

Supplementary Figure S6. Motor responses sampled from the left and right EBA separately. An analysis between groups (random effects GLM, n =17, p < 0.05 corrected) shows significant differences for movements of the right foot, right hand, left foot, and left hand in both hemispheres. Error bars represent the standard error. Large asterisks indicate between-group significance, small asterisks indicate within-group significance against the baseline. NS, non-significant. (* < 0.05, ** < 0.005).

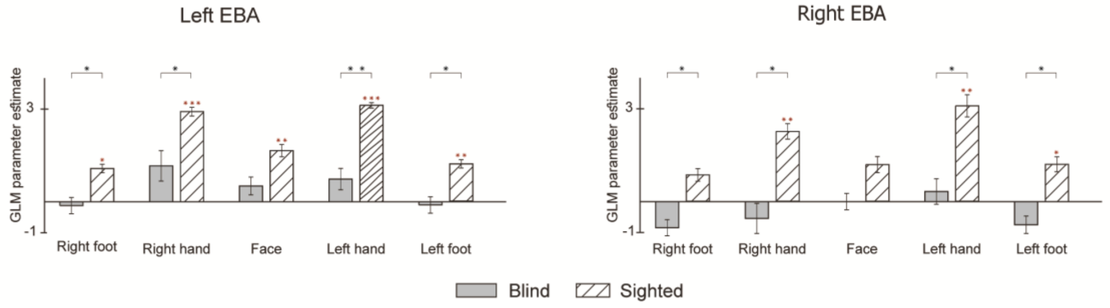

Supplementary Figure S7. M1 responses are comparable between blind and sighted individuals. There are no significant group differences in Right M1 activity (right hemisphere) for all unilateral and face movements (Table S4). ROI analysis of left M1 produced similar results (Fig. 2). The sighted and blind groups show similar somatotopic organization in S1 and M1 (right hemisphere). Large asterisks indicate between-group significance, small asterisks indicate within-group significance against the baseline. NS, non-significant. (* < 0.05, ** < 0.005, *** < 0.0005).

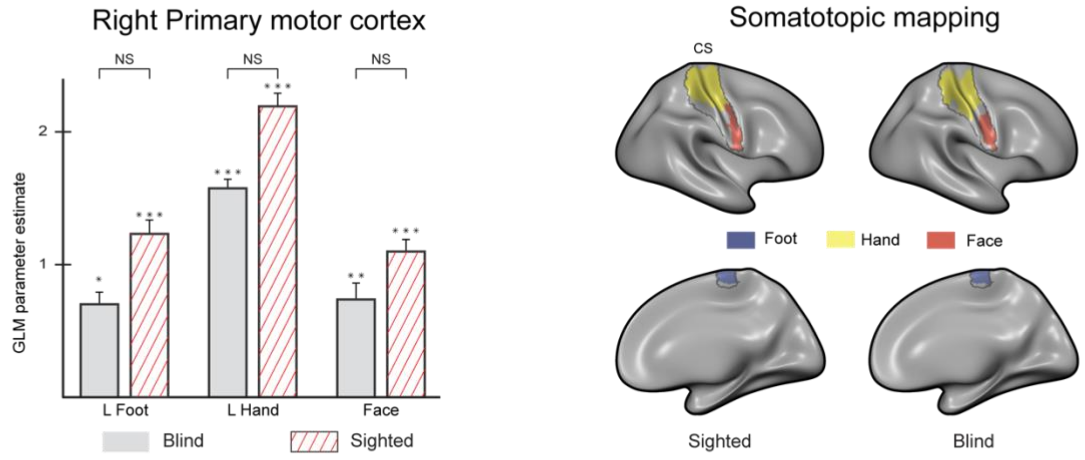

Supplementary Figure S8. Seed-to-seed analysis of connectivity strength from the EBA to sensorimotor and visual seeds in the left and right hemispheres. In the blind (n = 9) and sighted (n = 20), resting-state connectivity to visual areas was significantly higher than the baseline in each hemisphere. Connectivity to sensorimotor regions was stronger in the sighted compared to the blind group in both hemispheres (Random effects GLM, p < 0.05, n = 29). Large asterisks indicate between-group significance, small asterisks indicate within-group significance against the baseline. NS, non-significant. (* < 0.05, ** < 0.005, *** < 0.0005).

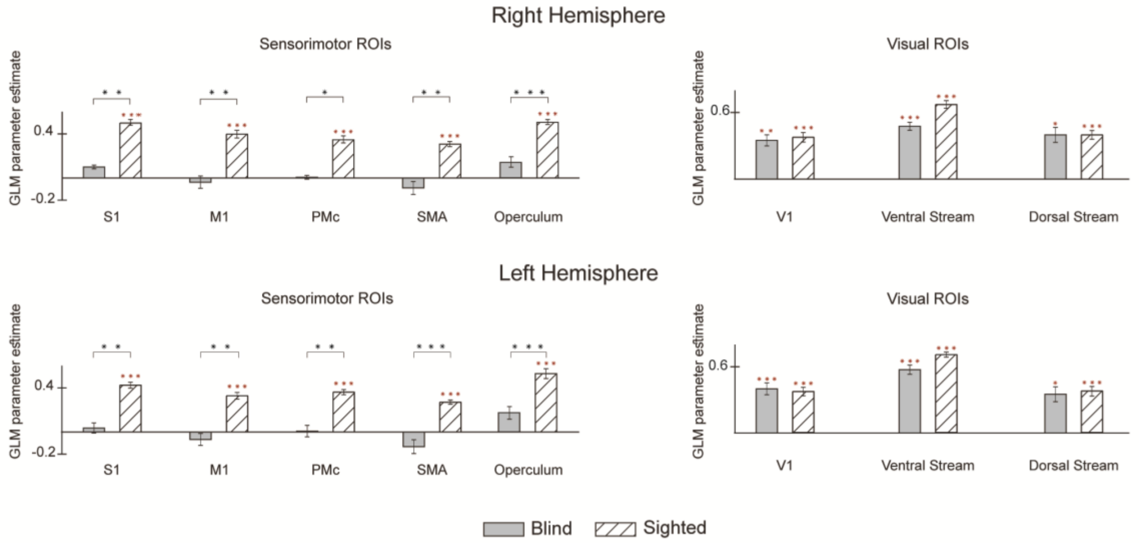

Supplement: Supplementary file 1 [file Data_Sheet_1.pdf]
